# Supplementary material for: Photoreactivity of Hair Melanin from Different Skin Phototypes—Contribution of Melanin Subunits to the Pigments Photoreactive Properties
Source: Int J Mol Sci. 2021 Apr 24;22(9):4465. doi: 10.3390/ijms22094465 (PMC8123205; doi:10.3390/ijms22094465)
Supplement: Supplementary file 1 [file ijms-22-04465-s001.zip › ijms-1189306-supplementary.pdf]

Supplementary material for:

Photoreactivity of hair melanin from different skin phototypes – contribution of melanin subunits to the pigments photoreactive properties

Table S1. Chemical analysis of isolated pigments. Numerical values of the ratios of examined markers for melanosomes and melanin nanoaggregates of the studied melanins. Data represent mean ± SEM.

Melanosomes	A650/A500 x10	PTCA/A500 (µg)	AHPs/A500 (µg)	TTCA/A500 (µg)
Red	1.79 ± 0.21	0.47 ± 0.17	1.67 ± 0.97	1.67 ± 0.27
Blond	2.48 ± 0.48	0.94 ± 0.13	0.66 ± 0.64	0.81 ± 0.61
Chestnut	2.85 ± 0.13	1.04 ± 0.12	0.03 ± 0.02	0.25 ± 0.08
Black	2.73 ± 0.13	1.29 ± 0.25	0.02 ± 0.004	0.12 ± 0.03

Table S2. Chemical analysis of isolated pigments. Numerical values of the ratios of examined markers for melanosomes and melanin nanoaggregates of the studied melanins. Data represent mean ± SEM.

Melanin nanoaggregates	A650/A500 ×10	PTCA/A500 (µg)	AHPs/A500 (µg)	TTCA/A500 (µg)
Red	0.93 ± 0.15	0.18 ± 0.05	2.08 ± 0.95	1.15 ± 0.61
Blond	0.65 ± 0.17	0.20 ± 0.11	0.73 ± 0.13	0.81 ± 0.20
Chestnut	0.90 ± 0.30	0.86 ± 0.08	0.84 ± 0.46	0.15 ± 0.11
Black	3.09 ± 0.02	0.34 ± 0.01	0.01 ± 0.002	0.04 ± 0.005

Table S3. Chemical analysis of isolated pigments. Numerical values of the ratios of examined markers for melanosomes of the studied melanins. Data represent mean ± SEM.

Melanosomes	PTCA/PDCA	TTCA/4-AHP	4-AHP/3-AHP ×10	AHPs/PTCA ×10	TTCA/PTCA ×10
Red	3.31 ± 1.60	2.98 ± 1.22	10.46 ± 2.72	52.00 ± 22.50	51.61 ± 23.80
Blond	13.12 ± 9.67	12.00 ± 16.76	6.54 ± 2.96	21.20 ± 21.10	23.76 ± 22.50
Chestnut	11.12 ± 0.50	27.43 ± 10.50	9.32 ± 5.71	0.37 ± 0.17	2.38 ± 0.54
Black	24.37 ± 7.38	29.66 ± 14.60	6.74 ± 1.39	0.13 ± 0.04	1.05 ± 0.37

Table S4. Chemical analysis of isolated pigments. Numerical values of the ratios of examined markers for melanosomes of the studied melanins. Data represent mean ± SEM.

Melanin Nanoaggregates	PTCA/PDCA	TTCA/4-AHP	4-AHP/3-AHP ×10	AHPs/PTCA ×10	TTCA/PTCA ×10
Red	1.19 ± 0.16	1.66 ± 0.68	0.72 ± 0.20	10.42 ± 2.48	5.54 ± 1.45
Blond	0.79 ± 0.17	3.07 ± 0.70	0.61 ± 0.12	9.48 ± 6.16	9.64 ± 5.56
Chestnut	3.84 ± 0.82	0.59 ± 0.48	1.08 ± 0.64	1.04 ± 0.62	0.17 ± 0.11
Black	1.44 ± 0.44	5.38 ± 0.01	1.19 ± 0.06	0.04 ± 0.01	0.11 ± 0.02

Table S5. Values of initial intensities of oxygen consumption for melanosomes and melanin nanoaggregates from different hair samples. Data represent mean ± s.d.

Type of hair melanin	Melanosomes		Nanoaggregates	
	365 nm (mM/s)	445 nm (mM/s)	365 nm (mM/s)	365 nm (mM/s)
Red	(5.20 ± 0.41) × 10^{-5}	(8.59 ± 0.72) × 10^{-6}	(4.90 ± 0.34) × 10^{-5}	(1.20 ± 0.05) × 10^{-5}
Blond	(8.07 ± 0.70) × 10^{-5}	(1.73 ± 0.12) × 10^{-5}	(8.19 ± 0.71) × 10^{-3}	(9.20 ± 0.84) × 10^{-4}
Chestnut	(1.24 ± 0.06) × 10^{-4}	(2.12 ± 0.13) × 10^{-5}	(4.80 ± 0.39) × 10^{-3}	(4.52 ± 0.38) × 10^{-4}
Black	(3.48 ± 0.21) × 10^{-5}	(6.10 ± 0.43) × 10^{-6}	(3.20 ± 0.26) × 10^{-5}	(7.21 ± 0.58) × 10^{-6}

Table S6. Values of initial velocities of DMPO-OOH formation by melanosomes and melanin nanoaggregates from different hair samples. Data represent mean ± s.d.

Type of hair melanin	Melanosomes		Nanoaggregates	
	365 nm (a.u./s)	445 nm (a.u./s)	365 nm (a.u./s)	445 nm (a.u./s)
Red	(7.40 ± 0.40) × 10^{-4}	(7.96 ± 0.57) × 10^{-5}	(4.58 ± 0.28) × 10^{-3}	(9.84 ± 0.69) × 10^{-4}
Blond	(8.23 ± 0.56) × 10^{-4}	(2.60 ± 0.16) × 10^{-4}	(6.95 ± 0.38) × 10^{-2}	(7.71 ± 0.55) × 10^{-3}
Chestnut	(1.82 ± 0.15) × 10^{-3}	(2.31 ± 0.16) × 10^{-4}	(2.62 ± 0.18) × 10^{-2}	(1.12 ± 0.09) × 10^{-3}
Black	(4.90 ± 0.34) × 10^{-4}	(6.65 ± 0.50) × 10^{-5}	(3.37 ± 0.27) × 10^{-3}	(7.22 ± 0.36) × 10^{-4}

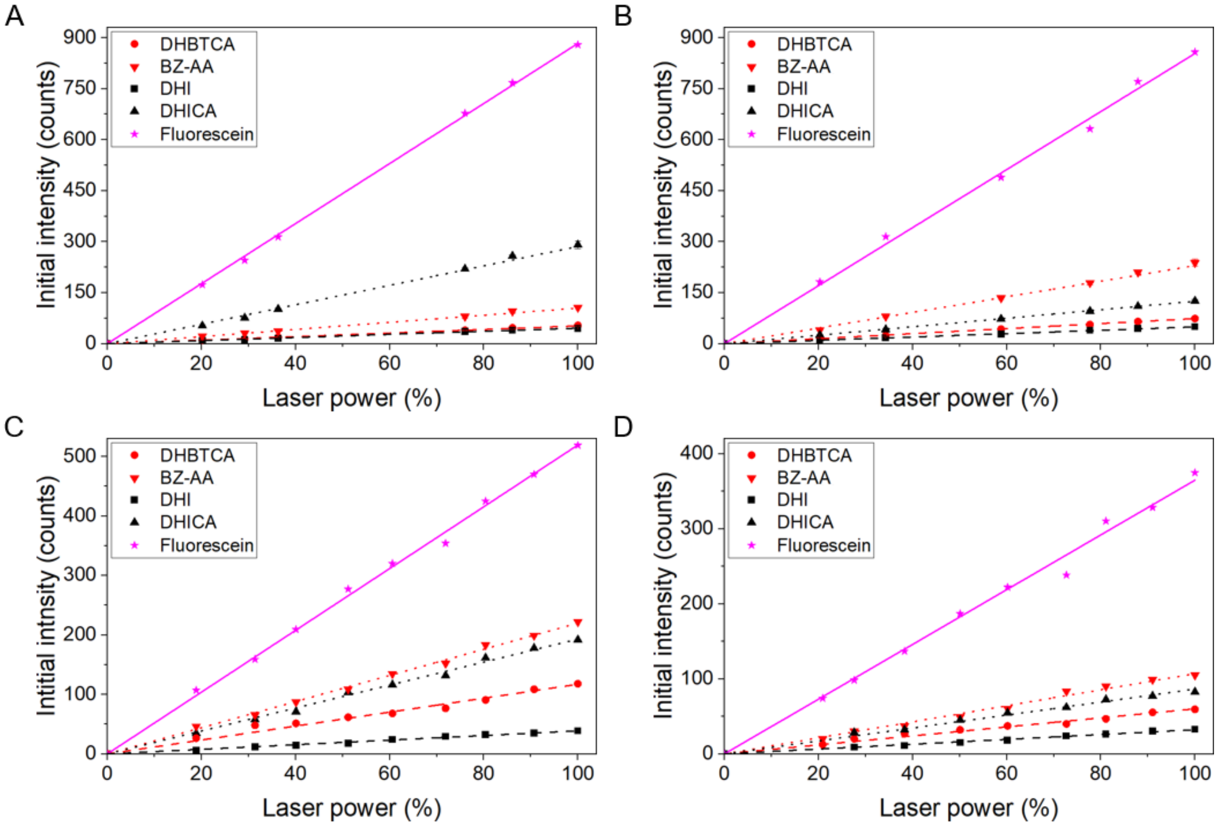

Figure S1. Determination of quantum yield of singlet oxygen photogeneration of melanin monomers at: 300 nm laser excitation (A), 332 nm laser excitation (B), 365 nm laser excitation (C) and 445 nm laser excitation (D). Magenta in A, B and D represents fluorescein as a reference sample, whereas magenta in C represent proflavine used as a standard.
